# Brazilian Registry of Sjögren’s Disease (BRAS): a full picture of Sjögren’s disease in Brazil

**DOI:** 10.1186/s42358-025-00481-4

**Published:** 2025-10-27

**Authors:** Valéria Valim, Samira Tatiyama Miyamoto, Fabiola Reis de Oliveira, Érica Vieira Serrano, Laura Caldas dos Santos, Roberta de Almeida Pernambuco, Simone Appenzeller, Juliana Markus, Leandro Augusto Tanure, Maria Lúcia Lemos Lopes, Rafael Coradin, Aysa César Pinheiro, Vanessa Hax, Aiessa Zanchett Fedrigo, Sandra Lúcia Euzébio Ribeiro, Karina Gatz Capobianco, Giovanna Sant’Ana Petterle, Alisson Pugliesi, Débora Cerqueira Calderaro, Ketty Lysie Libardi Lira Machado, Paula Regina Toche dos Santos, Nathalia de Carvalho Sacilotto, Anna Maria de Senna Migueletto, Diego Ustárroz Cantali, Vitalina de Souza Barbosa, Mateus Maia Marzola, Eduardo Melani Rocha, Ricardo Machado Xavier, Virgínia Fernandes Moça Trevisani

**Affiliations:** 1 Hospital Universitário Cassiano Antônio Moraes, Programa de Pós-graduação em Saúde Coletiva, Departamento de Clínica Médica, Universidade Federal do Espírito Santo, Vitória, ES Brazil; 2 Departamento de Educação Integrada em Saúde, Universidade Federal do Espírito Santo, Vitória, ES Brazil; 3 Hospital das Clínicas da Faculdade de Medicina de Ribeirão Preto da USP, Ribeirão Preto, SP Brazil; 4 Hospital Universitário Cassiano Antônio Moraes, Universidade Federal do Espírito Santo, Vitória, ES Brazil; 5 Universidade Federal de São Paulo, São Paulo, SP Brazil; 6 Instituto de Assistência Médica ao Servidor Público Estadual, São Paulo, SP Brazil; 7 Hospital das Clínicas da Universidade Estadual de Campinas, Campinas, SP Brazil; 8 Hospital de Clínicas da Universidade Federal de Uberlândia, Uberlândia, MG Brazil; 9 Hospital das Clínicas da Universidade Federal de Minas Gerais, Belo Horizonte, MG Brazil; 10 Irmandade da Santa Casa de Misericórdia de Porto Alegre, Universidade Federal de Ciências da Saúde de Porto Alegre, Porto Alegre, RS Brazil; 11 Hospital das Clínicas da Universidade Federal de Pernambuco, Recife, PE Brazil; 12 Hospital de Clínicas de Porto Alegre da Universidade Federal do Rio Grande do Sul, Porto Alegre, RS Brazil; 13 Hospital Universitário Evangélico Mackenzie, Curitiba, PR Brazil; 14 Universidade Federal do Amazonas, Manaus, AM Brazil; 15 Hospital Moinhos de Vento, Porto Alegre, RS Brazil; 16 Hospital Universitário Julio Müller, Universidade Federal de Mato Grosso, Cuiabá, MT Brazil; 17 Hospital São Lucas da Pontifícia Universidade Católica do Rio Grande do Sul, Porto Alegre, RS Brazil; 18 Hospital das Clínicas, Universidade Federal de Goiás, Goiás, GO Brazil; 19 Departamento de Reumatologia, Hospital de Clínicas de Porto Alegre, Universidade Federal do Rio Grande do Sul, Porto Alegre, RS Brazil; 20 Universidade Federal de São Paulo e Universidade de Santo Amaro, UNISA, São Paulo, SP Brazil; 21 Hospital Universitário Cassiano Antônio Moraes, Universidade Federal do Espírito Santo (HUCAM-UFES/EBSERH), Av. Mal. Campos, 1355, Santos Dumont, Vitória, Espírito Santo CEP 29041-295 Brazil

**Keywords:** Sjögren's disease, Registry, Epidemiology, Cohort, Prevalence

## Abstract

Cohort studies are essential for elucidating disease progression, guiding research priorities, and identifying gaps in diagnosis and treatment. This manuscript presents the protocol and preliminary findings of the Brazilian Registry on Sjögren’s Disease (BRAS), a national, prospective cohort supported by the Brazilian Society of Rheumatology (SBR). BRAS aims to collect comprehensive data on patients with Sjögren’s Disease (SjD) who meet the 2002 AECG and/or 2016 ACR-EULAR classification criteria, fostering high-quality research initiatives. Data collection is conducted via REDCap and includes demographic, laboratory, and clinical parameters such as disease activity (ESSDAI), damage (SSDDI), comorbidities, cardiovascular risk (Framingham score), labial salivary gland biopsy, salivary gland ultrasound, and therapeutic approaches. Patient-reported outcome measures (PROMs) include ESSPRI, PROFAD, HADS, ESE, IPAQ-SF, and EQ-5D. To date, 1,082 patients have been enrolled (mean age 55.3 ± 13.3 years; 96.6% women). Major findings include xerostomia (93%), xerophthalmia (92%), positive lip biopsy (83.6%), anti-Ro/SSA antibodies (77%), anti-La/SSB (43%), systemic manifestations (71.3%, ESSDAI 4.8 ± 5.8), and organ damage (86.9%, SSDDI 2.3 ± 1.6). Average disease duration was 7 ± 6.3 years with a diagnostic delay of 3.7 years. Treatments included corticosteroids (18%), hydroxychloroquine (46%), immunosuppressants (38%), and rituximab (6.5%). Common comorbidities were hypertension (34.9%), obesity (30.2%), dyslipidemia (29.7%), fibromyalgia (26%), osteoarthritis (24%), hypothyroidism (23.6%), diabetes (12.9%), and cancer (12.7%). Patients reported high symptom burden (ESSPRI 5.4 ± 2.4), anxiety (48%), depression (40%), low quality of life (63.6 ± 22.2), and insufficient physical activity (only 32.7% exercised regularly). Findings highlight the need for a multidisciplinary care program. The findings reveal diagnostic delays, limited healthcare access, high symptom burden, systemic involvement, poor mental health and highlight the need for a multidisciplinary care program. The next phase involves establishing a biorepository for biological samples.

**Clinical trial number** Not applicable.

## Background

Sjögren’s disease (SjD) is a chronic, immune-mediated systemic condition characterized by lymphocytic infiltration of exocrine glands, leading to xerostomia and xerophthalmia, and often accompanied by systemic manifestations. More than half of patients develop extraglandular features, which are associated with worse prognosis and increased risk of lymphoma [1, 2]. The estimated global prevalence of SjD without overlap with other autoimmune diseases is 43.03 per 100,000 individuals, with a female-to-male ratio of up to 14:1 [3]. In Brazil, the only available population-based prevalence study, conducted in a major metropolitan area in the Southeast region, estimated a prevalence of 0.17% [4].

Despite advances in symptom management, there is no disease-modifying therapy capable of altering the natural course of SjD [5, 6]. Dryness, fatigue, and pain remain significant challenges, impacting patients’ health-related quality of life (HRQoL). Several studies have shown that HRQoL is markedly impaired in patients with SjD compared to healthy individuals and is primarily associated with symptoms rather than systemic activity [7]. Longitudinal data are essential to better understand the evolution of symptoms, systemic activity, and their impact on HRQoL.

The development of standardized evaluation tools, such as the ESSDAI and ESSPRI proposed by the EULAR Sjögren’s Task Force [8], and the adoption of the ACR/EULAR 2016 classification criteria [9], have enabled better characterization and comparison of patient cohorts across different regions [10, 11]. However, the clinical expression of SjD is known to vary according to geographic, genetic, and environmental factors [5, 6, 12], which underscores the importance of collecting data from different countries. In the past decade, several national and international cohorts have been established to better characterize the disease in distinct populations, including the Big Data Sjögren Project [13, 14], the Sjögren’s International Collaborative Clinical Alliance (SICCA) [15, 16], the HarmonicSS project [17, 18], the UK Primary Sjögren’s Syndrome Registry (UKPSSR) [19, 20], the French ASSESS cohort [21], and the SJOGREN-SER in Spain [22]. These initiatives have provided valuable insights into the phenotypic diversity and epidemiological patterns of SjD and reinforce the need for robust national data to guide local clinical practice and contribute to global collaborative efforts.

In this context, the Brazilian Society of Rheumatology, through its Committee on Sjögren’s Disease, launched the Brazilian Registry of Sjögren’s Disease (BRAS). This prospective, multicenter initiative aims to characterize the clinical and epidemiological features of SjD in Brazil, support clinical decision-making, and promote national and international collaborations.

This manuscript presents the BRAS registry protocol, detailing its methodology, objectives, and preliminary descriptive findings.

## Methods

**Fig. 1:**
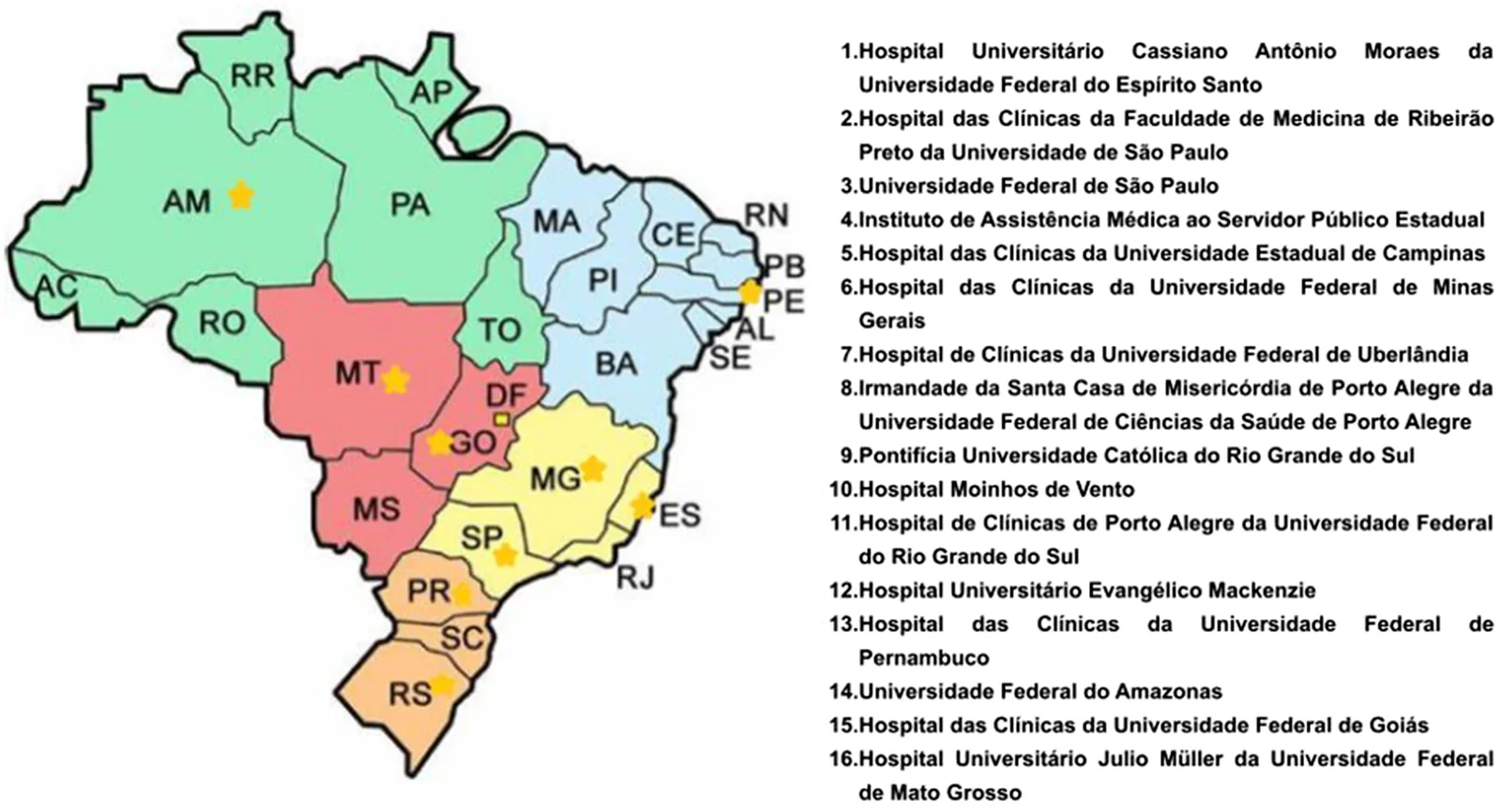
Brazilian Registry on Sjögren’s Disease (BRAS) centers in all regions of Brazil

**Fig. 2:**
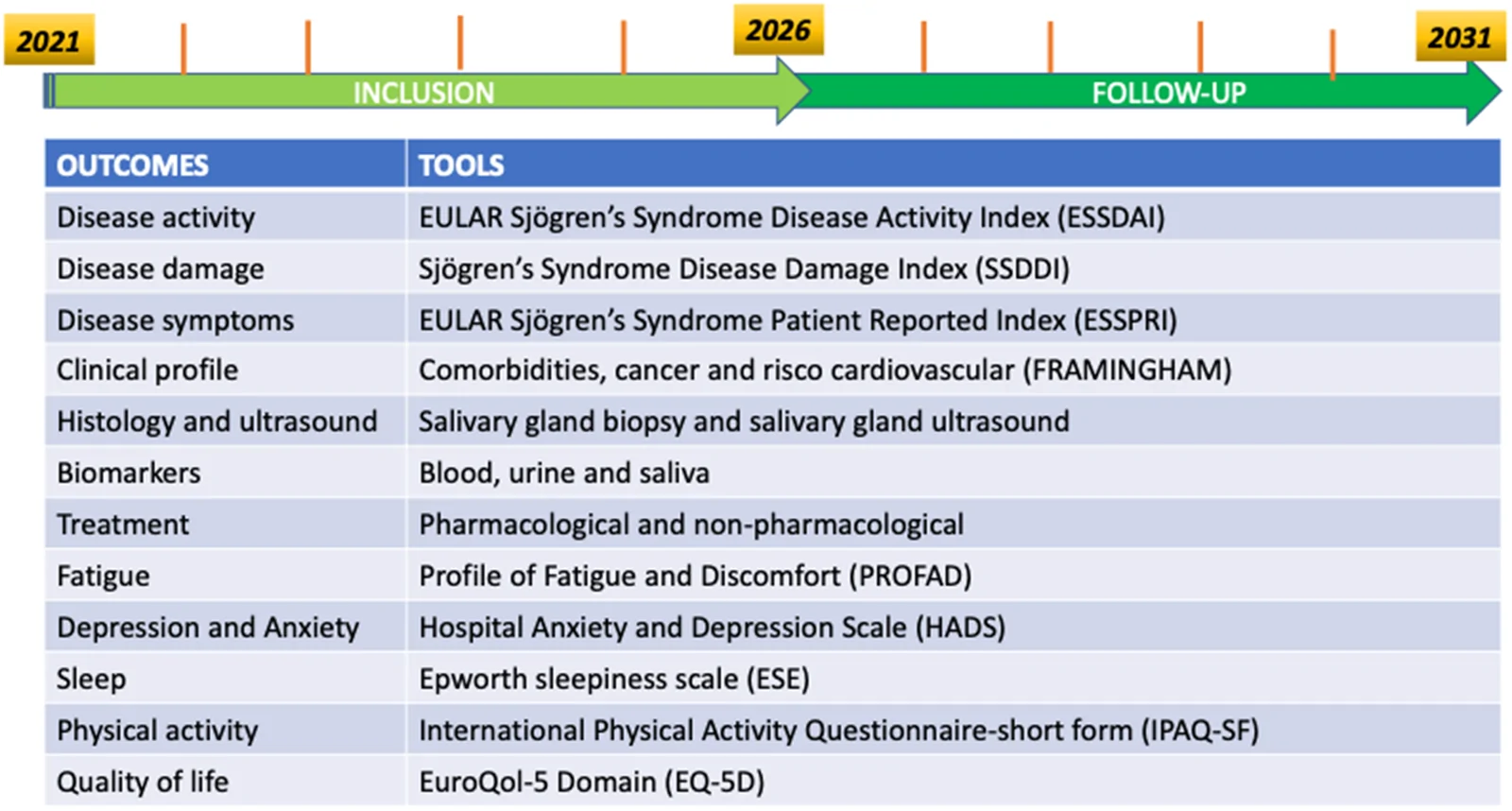
Outcomes evaluated in the Brazilian Registry on Sjögren’s Disease (BRAS) over 10-years of follow-up

The BRAS cohort is a prospective national registry. To date, 16 centers from all Brazilian regions are engaged in the collaborative endeavor, with the potential for additional centers to join until 2026 (Fig. 1). Patients enrolled in the registry will be monitored for ten years (Fig. 2).

The study includes patients diagnosed with SjD with no limit on disease duration, who meet the classification criteria set forth by the American-European Consensus Group (AECG) of 2002 and/or 2016 ACR/EULAR [9]. Individuals with a prior diagnosis of other conditions manifesting with xerophthalmia and/or xerostomia are excluded. These conditions include, but are not limited to, C-virus hepatitis, acquired immunodeficiency syndrome (AIDS), head and neck radiotherapy in the past, graft versus host disease, sarcoidosis, and hyperIgG4-related disease, as well as SjD associated with other connective tissue diseases.

The overall aim of BRAS is to generate a set of Brazilian clinical epidemiological data on patients with SjD to promote high-quality clinical research. The BRAS has the following specific objectives: (1) To characterize the cluster and endotypes of the disease; (2) To assess the frequency of patients meeting the 2016 ACR-EULAR classification criteria for SjD; (3) To describe the disease activity index, symptom, severity and damage index. 4; To study the clinical correlation with histological and immunological data; 5. To describe salivary gland ultrasound findings and correlate them with disease activity, severity of symptoms and damage, histological and immunological data 6; To compare the clinical profile of patients from different Brazilian geographic regions and ethnicities with those from other countries; 7. To assess the relationship between symptoms (fatigue, pain, dryness, anxiety, depression, and sleepiness), disease activity, and physical activity level; 8. To assess cardiovascular risk; 9. To assess the prevalence of comorbidities and polypharmacy; 10. To study the mortality of SjD.

The registry is currently collecting data pertaining to demographics, clinical characteristics, laboratory results, and imaging findings. The Research Electronic Data Capture (REDCap) platform, an integrated web-based system, is being employed for the purposes of data entry.

Demographic data (self-reported), clinical, and laboratory data were collected, including disease activity (EULAR Sjögren’s Syndrome Disease Activity Index - ESSDAI) [8, 23, 24], disease damage (Sjögren’s Syndrome Disease Damage Index - SSDDI) [25], comorbidities, cardiovascular risk (Framingham), labial salivary gland biopsy, salivary gland ultrasound, and pharmacological and non-pharmacological treatment. Additionally, patient-reported outcome measures (PROMs) are being incorporated, including the EULAR Sjögren’s Syndrome Patient-Reported Index (ESSPRI) [26, 27], Profile of Fatigue and Discomfort (PROFAD) [28, 29], Hospital Anxiety and Depression Scale (HADS) [30, 31], Epworth sleepiness scale (ESE) [32, 33], International Physical Activity Questionnaire-short form (IPAQ-SF) [34], and EuroQol-5 Domain (EQ-5D) [35]. Comorbidities diagnosed using international current criteria and/or registered in medical records.

This study was conducted in accordance with the ethical principles outlined in the Declaration of Helsinki. The study was approved by the Research Ethics Committee of the Hospital Universitário Cassiano Antônio Moraes da Universidade Federal do Espírito Santo, under approval number 2.930.715 (CAAE: 99071018.3.1001.5071), in October 18th 2018. All research centers got approval from the local Ethics Committee and patients provided informed consent to participate in the study. Clinical trial number: not applicable.

The current analysis encompasses patients from 16 clinical centers, as detailed in Table 2. Descriptive analyses were conducted to characterize the demographic and clinical data.

## Results

The mean age of the 1,082 SjD patients was 55.3 ± 13.3 years, with 96.5% being women. Most were white (58.7%), employed or retired (37% and 31%), and married (57.8%) (Table 1). Xerostomia and xerophthalmia were present in 93% and 91.6% of patients, respectively. Anti-Ro/SSA was detected in 77%, and anti-La/SSB in 42.5%. Among the 60.6% who underwent labial biopsy, 83.6% tested positive (Table 2). Additionally, 96.9% met the 2002 AECG and 98% the 2016 ACR-EULAR classification criteria. The mean disease duration was 7 ± 6.3 years, with an average diagnostic delay of 3.7 ± 5.1 years.

**Table 1:** Demographic characteristics of the Sjögren’s disease patients included in the Brazilian Registry of Sjögren’s Disease (BRAS)

	*n*	Mean (SD) or *n* (%)
Age, years, mean (SD)	977	55.3 (13.3)
Gender, n (%)	1082	
Female		1045 (96.6)
Male		37 (3.4)
Ethnicity, n (%)	1078	
Caucasian		633 (58.7)
African		75 (7.0)
Mixed		341 (31.6)
Asian		15 (1.4)
Indigenous		4 (0.4)
Occupation status, n (%)	1066	
Employed		394 (37.0)
Unemployed		79 (7.4)
Housewife		236 (22.1)
Retired		335 (31.4)
Disability benefits		20 (1.9)
Student		2 (0.2)
Marital status, n (%)	997	
Married		576 (57.8)
Single		177 (17.8)
Divorced		235 (23.6)
Time of disease, years, mean (SD)	838	7.0 (6.3)
Delay in diagnosis, years, mean (SD)	760	3.7 (5.1)
2002 AECG, n (%)	871	844 (96.9)
2016 ACR-EULAR, n (%)	864	847 (98.0)

**Table 2:** Criteria for Sjögren’s disease classification according to American-European consensus group 2022 or ACR-Eular 2016 and immunological biomarkers of the Sjögren’s disease patients included in the Brazilian Registry of Sjögren’s Disease (BRAS)

	Performed *n*	Mean (SD) or Positive, *n* (%)	Not performed *n* (%)
**Classification criteria**			
Dry mouth over 3 months, n (%)	617	573 (92.9)	-
Unstimulated salivary flow, positive, n (%)	484	380 (78.5)	143 (22.8)
Unstimulated salivary flow, mean (SD)	571	0.1 (0.2)	-
Focal lymphocytic sialodenitis, n (%)	433	362 (83.6)	180 (39.4)
Focus score, mean (SD)	473	1.9 (1.3)	-
Sialography, n (%)	40	2 (5)	578 (93.5)
Scintigraphy, n (%)	167	132 (79)	453 (73.1)
Dry eyes over 3 months, n (%)	619	567 (91.6)	-
Schirmer test, positive, n (%)	596	454 (76.2)	19 (3.1)
Schirmer test, right eye, mean (SD)	682	7.6 (9.6)	32 (4.5)
Schirmer test, left eye, mean (SD)	685	7.9 (9.6)	30 (4.2)
Van Bijesterveld, n (%)	131	77 (58.8)	593 (85.3)
Van Bijesterveld, right eye, mean (SD)	102	5.2 (3.1)	593 (85.3)
Van Bijesterveld, left eye, mean (SD)	101	5.1 (3.1)	596 (85.5)
Ocular Staining (OSS), n (%)	164	51 (31.1)	558 (78.6)
Ocular Staining (OSS), right eye, mean (SD)	152	3.2 (3.3)	558 (78.6)
Ocular Staining (OSS), left eye, mean (SD)	154	3.2 (3.3)	556 (78.3)
Tear Break Up time (BUT), n (%)	289	273 (94.5)	433 (61.2)
Tear Break Up time (BUT), right eye, mean (SD)	274	3.9 (2.7)	433 (61.2)
Tear Break Up time (BUT), left eye, mean (SD)	276	3.9 (2.8)	434 (61.1)
Anti-Ro, positive, n (%)	706	545 (77.2)	10 (1.4)
Anti-La, positive, n (%)	577	245 (42.5)	51 (8.1)
**Immunological biomarkers**			
ANA, positive, n (%)	844	694 (82.2)	13 (1.5)
RF, positive, n (%)	801	370 (46.2)	48 (5.7)
Cryoglobulins, positive, n (%)	832	19 (3.7)	315 (37.9)
C3, mg/dL, mean (SD)	567	76.8 (63.0)	23 (3.8)
C3 ≤ 90 mg/dL, yes, n (%)	568	70 (12.3)	
C4, mg/dL, mean (SD)	582	17.2 (17.6)	22 (3.6)
C4 ≤ 10 mg/dL, yes, n (%)	585	47 (8.0)	
IgG, mg/dL, mean (SD)	276	1541.7 (543.2)	169 (38.0)
ESR, mm/h, mean (SD)	590	30.7 (27.6)	79 (11.8)
CRP, mg/dL, mean (SD)	586	4.0 (9.6)	80 (12.0)
Gammaglobulin, g/dL, mean (SD)	521	1.5 (0.7)	98 (15.8)

ANA = Antinuclear Antibodies, RF = Rheumatoid Factor, ESR = Erythrocyte Sedimentation Rate, CRP = C-Reactive Protein

Cardiovascular risk factors and multimorbidities were common: hypertension (34.9%), obesity (30.2%), dyslipidemia (29.7%), fibromyalgia (26%), osteoarthritis (24%), hypothyroidism (23.6%), diabetes (12.9%), and cancer (12.7%). The occurrence of non-Hodgkin’s lymphoma was 3%. (Table 3).

**Table 3:** Comorbidities of the Sjögren’s disease patients included in the Brazilian Registry of Sjögren’s Disease (BRAS)

	*n*	Mean (SD) or Positive, *n* (%)
Body mass index, kg/m^2^, mean (SD)	701	27.4 (5.6)
Body mass index ≥ 30, n (%)	701	212 (30.2)
Smoking, n (%)	846	26 (3.1)
Framingham score, mean (SD)	852	4.2 (5.9)
Framingham percentage, mean (SD)	838	4.2 (5.4)
Comorbidities, n (%)		
Fibromyalgia	722	189 (26.2)
Regional pain syndrome	733	166 (22.6)
Diabetes	851	110 (12.9)
Hypertension	853	298 (34.9)
Dyslipidemia	844	251 (29.7)
Acute myocardial infarction	849	27 (3.2)
Stroke	671	16 (2.4)
Chronic arterial obstruction	846	12 (1.4)
Lymphoma	733	22 (3.0)
Skin cancer	828	23 (2.8)
Other cancer	829	57 (6.9)
Hypothyroidism	738	174 (23.6)
Hepatitis B	715	15 (2.1)
Hashimoto’s thyroiditis	653	91 (13.9)
Chronic renal failure	738	37 (5.0)
Osteoporosis	644	109 (16.9)
Osteoarthritis	732	175 (23.9)

Patients frequently exhibited systemic manifestations and had moderate-to-high ESSDAI scores at diagnosis, which decreased over time (initial ESSDAI vs. current ESSDAI = 8.3 ± 7.3 vs. 4.8 ± 5.8, *p* = 0.001). At the diagnosis swelling of the parotid, salivary, and/or lacrimal glands was identified in 34.4% patients, arthritis in 58.6%, cutaneous vasculitis in 11.7%, peripheral neuropathy in 9.1%, and interstitial lung disease in 13.3%. This may explain the high prevalence of disease-related damage in 86.9% (SSDDI = 2.3 ± 1.6) and also the use of medications for disease control: corticosteroids (18%), hydroxychloroquine (46%), immunosuppressants (38%), and rituximab (6.5%) (Tables 4 and 5).

Patients reported a high symptom burden (ESSPRI 5.4 ± 2.4), anxiety (48%), depression (40%), poor quality of life (63.6 ± 22.2), and low physical activity, with only 32.7% exercising regularly (Table 6).

**Table 4:** Medications in use by Sjögren’s disease patients included in the Brazilian Registry of Sjögren’s Disease (BRAS)

	*n*	Yes, *n* (%)
Medications, n (%)		
Pilocarpine	503	40 (8.0)
Cevimeline	503	7 (1.4)
Acetylcysteine	501	12 (2.4)
Linseed oil	495	240 (48.5)
Saliva substitute	493	53 (10.8)
Tear substitute	494	308 (62.3)
Corticosteroids (≤ 20 mg/d)	494	77 (15.6)
Corticosteroids (> 20 mg/d)	498	10 (2.0)
Hydroxychloroquine	484	223 (46.1)
Methotrexate, n (%)	501	96 (19.2)
Leflunomide, n (%)	505	31 (6.1)
Azathioprine, n (%)	483	61 (12.6)
Cyclophosphamide, n (%)	501	1 (0.2)
Mycophenolate mofetil, n (%)	504	23 (4.6)
Immunoglobulin, n (%)	502	3 (0.6)
Rituximab, n (%)	493	32 (6.5)
Abatacept, n (%)	503	2 (0.4)
NSAID, n (%)	497	7 (1.4)
Analgesic, n (%)	498	37 (7.4)
Opioid, n(%)	498	16 (3.2)
Antidepressant, n (%)	488	153 (31.4)
Antihypertensive, n(%)	492	161 (32.7)
Anticonvulsant, n (%)	502	42 (8.4)
Thyroxine, n (%)	503	106 (21.1)
Sinvastatin, n (%)	500	109 (21.8)
Antidiabetic, n (%)	502	48 (9.6)
Insulin, n (%)	500	8 (1.6)
Proton pump inhibitor, n (%)	496	78 (15.7)
Bisphosphonate, n (%)	501	47 (9.4)
Vitamin D, n (%)	499	223 (44.7)
Calcium, n (%)	492	119 (24.2)
Folic acid, n (%)	497	53 (10.7)

NSAID = Non-Steroidal Anti-Inflammatory Drug

**Table 5:** Comparison of ESSDAI in the beginning with the current ESSDAI of Sjögren’s disease patients included in the Brazilian Registry of Sjögren’s Disease (BRAS)

	At diagnosis Yes, *n* (%)	Current Yes, *n* (%)	At diagnosis mean (SD)	Current mean (SD)	*p*-value
	*n* = 840	*n* = 798	*n* = 840	*n* = 798	
Total ESSDAI	-	-	8.3 (7.3) (*n* = 840)	4.8 (5.8) (*n* = 798)	< 0.001
Constitutional	139 (16.7)	36 (4.6)	0.6 (1.4)	0.2 (0.7)	< 0.001
Lymphadenopathy	109 (13.0)	67 (8.7)	0.7 (1.9)	0.4 (1.5)	< 0.001
Glandular	287 (34.4)	139 (17.7)	0.9 (1.3)	0.4 (0.9)	< 0.001
Articular	491 (58.6)	309 (39.5)	1.6 (1.7)	1.0 (1.4)	< 0.001
Cutaneous	97 (11.7)	31 (3.8)	0.6 (1.8)	0.2 (1.1)	< 0.001
Pulmonary	111 (13.3)	99 (12.5)	1.3 (3.6)	1.0 (2.9)	0.002
Renal	35 (4.1)	26 (3.3)	0.4 (1.3)	0.3 (1.7)	0.138
Muscular	12 (1.4)	10 (1.2)	0.1 (1.3)	0.1 (1.2)	0.662
Peripheral Nervous System	76 (9.1)	48 (6.1)	0.8 (2.9)	0.5 (2.1)	0.001
Central Nervous System	30 (3.6)	13 (1.6)	0.4 (2.4)	0.2 (1.6)	0.004
Haematological	151 (18.2)	94 (12.5)	0.5 (1.1)	0.3 (0.9)	0,001
Biological	318 (38.5)	226 (30.5)	0.5 (0.7)	0.4 (0.6)	< 0.001
ESSDAI = 0, n (%)	81 (9.6)	229 (28.7)	-	-	< 0.001
ESSDAI ≥ 5, n (%)	504 (60.0)	299 (37.5)	-	-	< 0.001
ESSDAI ≥ 14, n (%)	191 (22.7)	86 (10.8)	-	-	< 0.001

ESSDAI = EULAR Sjögren’s Syndrome Disease Activity Index

**Table 6:** Symptoms, damage, mental health, sleepiness, quality of life and level of physical activity of the Sjögren’s disease patients included in the Brazilian Registry of Sjögren’s Disease (BRAS)

	*n*	Mean (SD) or Yes, *n* (%)
SSDDI, mean (SD)	844	2.3 (1.6)
SSDDI = 0, n (%)	844	105 (13.1)
ESSPRI total, mean (SD)	695	5.4 (2.4)
ESSPRI total ≥ 5, n (%)	695	449 (64.6)
ESSPRI dryness, mean (SD)	695	5.7 (2.9)
ESSPRI dryness ≥ 5, n (%)	695	489 (70.4)
ESSPRI fatigue, mean (SD)	695	5.5 (3.1)
ESSPRI fatigue ≥ 5, n (%)	695	424 (61.0)
ESSPRI pain, mean (SD)	694	5.0 (3.3)
ESSPRI pain ≥ 5, n (%)	694	406 (58.5)
PROFAD physical, mean (SD)	655	3.7 (2.1)
PROFAD physical ≥ 4, n (%)	655	330 (50.4)
PROFAD mental, mean (SD)	657	3.4 (2.3)
PROFAD mental ≥ 4, n (%)	657	310 (47.2)
EuroQoL 5D		
VAS, mean (SD)	632	63.6 (22.2)
Mobility, n (%)	652	
No problems in walking about		380 (58.3)
Some problems in walking about		262 (40.2)
Confined to bed		10 (1.5)
Self-care, n (%)	650	
No problems		516 (79.4)
Some problems with washing or dressing self		123 (18.9)
Unable to wash or dress self		11 (1.7)
Usual activities, n (%)	651	
No problems		342 (52.5)
Some problems		292 (44.9)
Unable		17 (2.6)
Pain/discomfort, n (%)	652	
No pain or discomfort		155 (23.8)
Moderate pain or discomfort		402 (61.7)
Extreme pain or discomfort		95 (14.6)
Anxiety/depression, n (%)	650	
No anxiety or depression		211 (32.5)
Moderately anxiety or depression		339 (52.2)
Extremely anxiety or depression		100 (15.4)
HADS - Anxiety, mean (SD)	611	8.6 (4.7)
HADS - Anxiety ≥ 9, n (%)	611	296 (48.4)
HADS - Depression, mean (SD)	611	7.6 (4.4)
HADS - Depression ≥ 9, n (%)	611	245 (40.1)
ESS, mean (SD)	688	7.9 (5.5)
ESS > 10, n (%)	688	190 (27.6)
IPAQ, MET-minutes/week, mean (SD)		
Total	523	2055.8 (3905.4)
Walking	453	619.7 (1058.3)
Moderate	386	1282.8 (1911.2)
Vigorous	190	1573.0 (3688.1)
IPAQ classification, n (%)	532	
Low		215 (40.4)
Moderate		210 (39.5)
High		107 (20.1)
Regular physical exercise, yes, n (%)	710	232 (32.7)
Physical therapy, n (%)	697	65 (9.3)

SSDDI = Sjögren’s Syndrome Disease Damage Index, ESSPRI = EULAR Sjögren’s Syndrome Patient-Reported Index, PROFAD = Profile of Fatigue and Discomfort, HADS = Hospital Anxiety and Depression Scale, ESS = Epworth Sleepiness Scale, IPAQ-SF = International Physical Activity Questionnaire-short form, EQ-5D = EuroQol-5 Domains

## Discussion

### The BRAS registry: A well-characterized cohort

The BRAS represents a well-characterized cohort, reflecting the demographic and serological profile consistent with other large international registries. The predominance of middle-aged women (median age 55 years; female: male ratio ~ 15:1), and autoantibody positivity rates—82.2% ANA, 46.2% RF, 77% anti-Ro/SSA, 42% anti-La/SSB—align with findings from the UK Primary Sjögren’s Syndrome Registry (UKPSSR) [19], the prospective ASSESS cohort [21], and the Spanish SER registry [22]. The higher proportion of Caucasians (58%) in BRAS compared to the general Brazilian population (43.5%) likely reflects demographic biases related to regional recruitment and healthcare access disparities, similar to ethnic variability reported in other multiethnic cohorts [14].

Registries such as BRAS and international consortia like the Big Data Sjögren Consortium [13, 14] and HarmonicSS [17] play an essential role in defining phenotypic heterogeneity and identifying environmental and genetic determinants of disease expression. Brazil’s unique ethnic diversity and environmental exposures render the BRAS registry a critical resource for broadening global understanding of Sjögren’s disease (SjD) in underrepresented populations.

### Comparison with international cohorts

The clinical and serological characteristics of BRAS participants are generally comparable to those reported in European and North American cohorts [11, 13, 14]. However, some distinctive features emerge. For instance, the proportion of patients reporting severe fatigue and pain in BRAS exceeds that observed in UKPSSR and ASSESS cohorts [19–21], which may reflect sociocultural factors, environmental exposures such as air pollution [14], or differences in healthcare infrastructure affecting symptom management.

Moreover, constitutional symptoms and mucocutaneous involvement appear more frequent in BRAS than in the SICCA and Spanish cohorts [15, 22], suggesting regional modifiers of disease phenotype possibly related to climate and pollution exposure, as previously highlighted in multinational analyses [14]. These differences underscore the importance of contextualizing SjD manifestations within geographical and environmental frameworks.

### Diagnosis and disease management

Despite persistent dryness complaints (> 90%), the average diagnostic delay of four years after symptom onset al.igns with international data highlighting challenges in early recognition of SjD [15, 19]. The incomplete application of standardized diagnostic tests—such as ophthalmologic staining (41.4%), Schirmer I test (91.6%), salivary flow assessment (60%), and salivary gland scintigraphy (27.2%)—reflects disparities in access to specialized evaluations, paralleling barriers described in other national registries [19, 22]. Labial salivary gland biopsy remains widely used (70%), consistent with clinical practice patterns globally.

These findings emphasize the need to improve healthcare provider training and infrastructure to ensure earlier diagnosis and uniform application of classification criteria, as well as to optimize patient outcomes.

### Comorbidities and cardiovascular risk

The prevalence of cardiovascular risk factors in BRAS—obesity, hypertension, diabetes, and dyslipidemia—is comparable to other cohorts [20, 36, 37], but the under-treatment of hypertension highlights a critical gap in comprehensive care. The high frequency of fibromyalgia (26.2%) and regional pain syndrome (22.6%) matches UKPSSR findings [38] and exceeds prevalence in the general population [39], suggesting that central sensitization and nociplastic pain contribute substantially to symptom burden in SjD.

The coexistence of autoimmune comorbidities such as Hashimoto’s thyroiditis and degenerative conditions like osteoarthritis further complicates clinical management, reinforcing the necessity for multidisciplinary approaches.

### Disease activity, treatment, and quality of life

Approximately 70% showed systemic manifestation and 40% of BRAS patients exhibit moderate-to-high systemic activity, reflected in ongoing use of corticosteroids (20%), hydroxychloroquine (46%), immunosuppressants (40%), and rituximab (6.5%). The observed decrease in ESSDAI scores over time may result from natural disease course or therapeutic interventions, consistent with longitudinal data from the ASSESS cohort [40]. Notably, a high proportion (86.9%) exhibit disease-related damage despite relatively short disease duration (~ 7 years), underscoring the progressive nature of SjD.

Patient-reported outcomes reveal a substantial symptom burden, with over 60% experiencing fatigue, nearly 59% reporting pain, and 70% dryness. Functional impairment and low physical activity levels contribute to poor mental health, as evidenced by high rates of anxiety (48%) and depression (41%), paralleling findings in other registries [7, 41].

### Need for a multidisciplinary care model

The clinical complexity and substantial morbidity documented in BRAS highlight an urgent need for structured, multidisciplinary care models integrating rheumatology, dentistry, ophthalmology, nutrition, physiotherapy, occupational therapy, and psychological support. Delayed diagnosis and limited access to comprehensive care remain barriers to optimal outcomes.

### Study limitations

Several limitations warrant consideration. The BRAS registry relies primarily on data from referral centers, which may introduce selection bias favouring more severe or complicated cases and limit generalizability to the broader Brazilian SjD population.

Due to a change in the data entry platform and the impact of the COVID-19 pandemic, the migration and completion of patient information into the new database have been delayed and are still ongoing. This explains why, although 1082 patients have been registered with demographic and classification data, many of them do not yet have their full clinical, laboratory, and patient-reported outcomes updated in the new system. Therefore, these data could not be included in the present analysis. The main objective of this manuscript is to describe the BRAS protocol and present preliminary findings.

Variability in diagnostic testing availability across centers and incomplete data capture may affect the accuracy of some findings. Additionally, the cross-sectional nature of the preliminary analysis precludes causal inferences. Future longitudinal follow-up will be critical to delineate disease trajectories, treatment effectiveness, and prognostic factors.

## Conclusion

The BRAS registry offers valuable insights into the clinical, serological, and psychosocial landscape of SjD in Brazil, revealing both parallels and divergences with international cohorts. Findings emphasize diagnostic delays, substantial symptom burden, high systemic involvement, high comorbidity prevalence, and mental health challenges, underscoring the imperative for comprehensive, multidisciplinary management strategies. Integration into global collaborative networks will enhance understanding and guide tailored interventions for Brazilian patients. The establishment of a biorepository represents a crucial next step to facilitate biomarker discovery and precision medicine approaches.

## Data Availability

The datasets used and/or analyzed during the current study are available from the corresponding author upon reasonable request.
